# Cytenamide–1,4-dioxane (2/1)

**DOI:** 10.1107/S1600536808018709

**Published:** 2008-06-28

**Authors:** Andrea Johnston, Alastair J. Florence, Francesca J. A. Fabbiani, Kenneth Shankland, Colin T. Bedford

**Affiliations:** a Solid-State Research Group, Strathclyde Institute of Pharmacy and Biomedical Sciences, The John Arbuthnott Building, University of Strathclyde, 27 Taylor Street, Glasgow G4 0NR, Scotland; b University of Göttingen, GZG, Department of Crystallography, Goldschmidtstrasse 1, D-37077 Göttingen, Germany; c ISIS Facility, Rutherford Appleton Laboratory, Chilton, Didcot, Oxon OX11 0QX, England; d University College London, Department of Chemistry, 20 Gordon Street, London WC1H 0AJ, England

## Abstract

In the crystal structure of the title compound [systematic name: 5*H*-dibenzo[*a*,*d*]cyclo­hepta­triene-5-carboxamide–1,4-dioxane (2/1)], 2C_16_H_13_NO·C_4_H_8_O_2_, the cytenamide mol­ecules form a hydrogen-bonded *R*_2_^2^(8) dimer. The solvent mol­ecule is located between two adjacent cytenamide dimers and forms N—H⋯O hydrogen bonds with one cytenamide mol­ecule from each dimer.

## Related literature

For details on experimental methods used to obtain this form, see: Davis *et al.* (1964 ▶); Florence *et al.* (2003 ▶); Florence, Johnston, Fernandes *et al.* (2006 ▶). For related literature on cytenamide, see: Florence, Bedford *et al.* (2008 ▶). For literature on related mol­ecules, see: Cyr *et al.* (1987 ▶); Fleischman *et al.* (2003 ▶); Florence, Johnston, Price *et al.* (2006 ▶); Florence, Leech *et al.* (2006 ▶); Bandoli *et al.* (1992 ▶); Harrison *et al.* (2006 ▶); Leech *et al.* (2007 ▶); Florence, Shankland *et al.* (2008 ▶). For other related literature, see: Etter (1990 ▶).
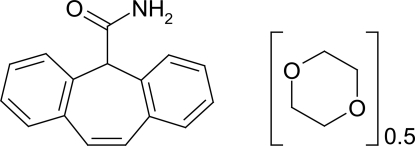


## Experimental

### 

#### Crystal data

2C_16_H_13_NO·C_4_H_8_O_2_
*M* *_r_* = 558.68
Monoclinic, 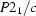

*a* = 24.0888 (7) Å
*b* = 5.6066 (2) Å
*c* = 21.1050 (6) Å
β = 90.313 (3)°
*V* = 2850.32 (15) Å^3^
*Z* = 4
Mo *K*α radiation
μ = 0.09 mm^−1^
*T* = 160 K
0.48 × 0.09 × 0.03 mm

#### Data collection

Oxford Diffraction Gemini S diffractometer
Absorption correction: multi-scan (*ABSPACK*; Oxford Diffraction, 2007 ▶) *T*_min_ = 0.84, *T*_max_ = 1.00 (expected range = 0.838–0.997)
23004 measured reflections
5125 independent reflections
3677 reflections with *I* > 2σ(*I*)
*R*_int_ = 0.057

#### Refinement

*R*[*F* ^2^ > 2σ(*F* ^2^)] = 0.068
*wR*(*F* ^2^) = 0.121
*S* = 1.08
5125 reflections
380 parameters
H-atom parameters constrained
Δρ_max_ = 0.47 e Å^−3^
Δρ_min_ = −0.42 e Å^−3^

### 

Data collection: *CrysAlis CCD* (Oxford Diffraction, 2007 ▶); cell refinement: *CrysAlis RED* (Oxford Diffraction, 2007 ▶); data reduction: *CrysAlis RED* and *SORTAV* (Blessing, 1997 ▶); program(s) used to solve structure: *SIR92* (Altomare *et al.*, 1994 ▶); program(s) used to refine structure: *CRYSTALS* (Betteridge *et al.*, 2003 ▶); molecular graphics: *ORTEP-3* (Farrugia, 1997 ▶) and *Mercury* (Macrae *et al.*, 2006 ▶); software used to prepare material for publication: *PLATON* (Spek, 2003 ▶).

## Supplementary Material

Crystal structure: contains datablocks I, global. DOI: 10.1107/S1600536808018709/tk2275sup1.cif

Structure factors: contains datablocks I. DOI: 10.1107/S1600536808018709/tk2275Isup2.hkl

Additional supplementary materials:  crystallographic information; 3D view; checkCIF report

## Figures and Tables

**Table 1:** Hydrogen-bond geometry (Å, °)

*D*—H⋯*A*	*D*—H	H⋯*A*	*D*⋯*A*	*D*—H⋯*A*
N1—H11⋯O2^i^	0.85	2.11	2.962 (3)	171
N1—H12⋯O4^i^	0.87	2.22	2.978 (3)	145
N2—H13⋯O1^ii^	0.87	1.95	2.823 (3)	177
N2—H14⋯O3^ii^	0.87	2.53	3.040 (3)	119

Symmetry codes: (i) 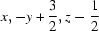; (ii) 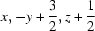.

**Fig. 1.**
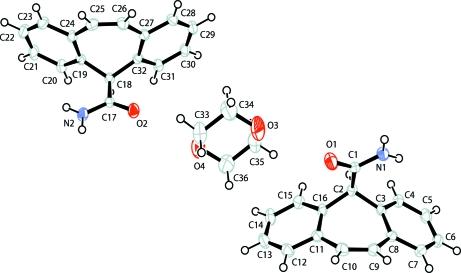
The molecular structure of (I), showing 50% probablility displacement ellipsoids.

**Fig. 2.**
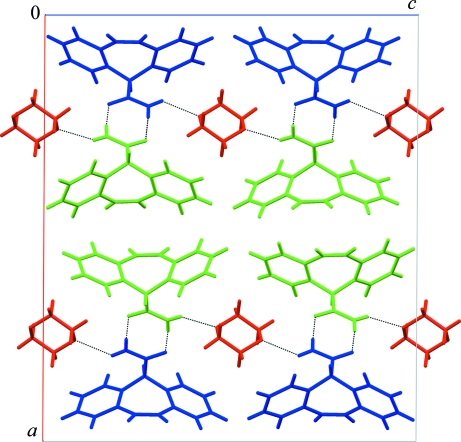
Packing diagram for (I) viewed down the b axis, showing the CYT R22(8) dimer motif further linked by N—H···O hydrogen bonds between CYT and dioxane molecules to form an infinite chain in the [001] direction. Molecules are coloured according to symmetry equivalence (CYT blue and green, dioxane molecules red) and hydrogen bonds are represented by dashed lines.

## supplementary crystallographic information

### Comment

Cytenamide (CYT) is an analogue of carbamazepine (CBZ), a dibenzazepine drug used to control seizures (Cyr *et al.*, 1987). CYT-dioxane hemisolvate was produced during an automated parallel crystallization study of CYT (Florence, Johnston, Fernandes *et al.*, 2006) as part of a wider investigation that couples automated parallel crystallization with crystal structure prediction methodology to investigate the basic science underlying the solid-state diversity of CBZ (Florence, Johnston, Price *et al.*, 2006; Florence, Leech *et al.*, 2006; Fleischman *et al.*, 2003) and its closely related analogues: CYT (Florence *et al.*, 2008*a*),10,11-dihydrocarbamazepine (Bandoli *et al.*, 1992; Harrison *et al.*, 2006; Leech *et al.*, 2007) and cyheptamide (Florence, Shankland *et al.*, 2008). The sample was identified as a new form using multi-sample foil transmission X-ray powder diffraction analysis (Florence *et al.*, 2003). Subsequent manual recrystallization from a saturated 1,4-dioxane solution by slow evaporation at 298 K yielded a sample suitable for single-crystal X-ray diffraction (Fig. 1).

The reported crystal structure is essentially isostructural with that of CBZ-dioxane solvate (2/1) (Florence, Johnston, Price *et al.*, 2006) and accordingly displays very similar packing arrangements. Specifically, the molecules crystallize with two CYT and one 1,4-dioxane molecules in the asymmetric unit. Pairs of CYT molecules form an *R*_2_^2^(8) dimer motif (Etter, 1990) *via* two N—H···O hydrogen bonds and a further two N—H···O contacts link CYT dimers with solvent molecules to form an infinite chain that extends in the c-direction (Table 1 & Fig. 2).

### Experimental

A sample of cytenamide was synthesized according to a modification of the published method (Davis *et al.*, 1964). A single crystal of (I) was grown from a saturated solution of cytenamide in 1,4-dioxane by isothermal solvent evaporation at 298 K.

### Refinement

Data were merged with *SORTAV* (Blessing, 1997) and a theta cut off of 25.0 ° was applied due to weak scattering. H-atoms were found on a difference Fourier map and were initially refined with soft restraints on the bond lengths and angles to regularize their geometry. The C-H distances are in the range 0.92 - 0.98 Å and *U*_iso_(H) = 1.2-1.5*U*_eq_(C). Atoms C12 C13 C14 and to some extent C15 suffer from large and prolate thermal ellipsoids. During refinement, the crystal was found to be twinned, according to the twin law expressed by the following matrix: 1 0 0.012, 0 - 1 0, 0 0 - 1 *i.e.* approximately about the *a* axis, giving rise to a twin component of *ca* 10%. Inclusion of the twin resulted in a reduction of the *R*-factor by *ca* 1%.

### Crystal data

**Table:**

2C_16_H_13_NO·C_4_H_8_O_2_	*F*_000_ = 1184
*M**_r_* = 558.68	*D*_x_ = 1.302 Mg m^−^^3^
Monoclinic, *P*2_1_/*c*	Mo *K*α radiation λ = 0.71073 Å
Hall symbol: -P 2ybc	Cell parameters from 4484 reflections
*a* = 24.0888 (7) Å	θ = 3–27º
*b* = 5.6066 (2) Å	µ = 0.09 mm^−^^1^
*c* = 21.1050 (6) Å	*T* = 160 K
β = 90.313 (3)º	Plate, colourless
*V* = 2850.32 (15) Å^3^	0.48 × 0.09 × 0.03 mm
*Z* = 4	

### Data collection

**Table:**

Area diffractometer	5125 independent reflections
Monochromator: graphite	3677 reflections with *I* > 2σ(*I*)
Detector resolution: 15.9745 pixels mm^-1^	*R*_int_ = 0.057
*T* = 160 K	θ_max_ = 25.2º
ω scans	θ_min_ = 2.6º
Absorption correction: multi-scan(ABSPACK; Oxford Diffraction, 2007)	*h* = −28→28
*T*_min_ = 0.84, *T*_max_ = 1.00	*k* = 0→6
23004 measured reflections	*l* = 0→25

### Refinement

**Table:**

Refinement on *F*^2^	Hydrogen site location: inferred from neighbouring sites
Least-squares matrix: full	H-atom parameters constrained
*R*[*F*^2^ > 2σ(*F*^2^)] = 0.068	Method = Modified Sheldrick *w* = 1/[σ^2^(*F*^2^) + (0.03*P*)^2^ + 2.31*P*], where *P* = [max(*F*_o_^2^,0) + 2*F*_c_^2^]/3
*wR*(*F*^2^) = 0.121	(Δ/σ)_max_ < 0.001
*S* = 1.08	Δρ_max_ = 0.47 e Å^−^^3^
5125 reflections	Δρ_min_ = −0.42 e Å^−^^3^
380 parameters	Extinction correction: None
Primary atom site location: structure-invariant direct methods	

### Fractional atomic coordinates and isotropic or equivalent isotropic displacement parameters (Å^2^)

**Table:**

	*x*	*y*	*z*	*U*_iso_*/*U*_eq_	
C1	0.30658 (11)	0.7244 (5)	0.22235 (12)	0.0230	
C2	0.34609 (11)	0.5089 (5)	0.22501 (12)	0.0212	
C3	0.38005 (11)	0.4805 (5)	0.16566 (12)	0.0223	
C4	0.36884 (12)	0.2923 (5)	0.12509 (12)	0.0258	
C5	0.39561 (13)	0.2692 (6)	0.06774 (13)	0.0325	
C6	0.43397 (12)	0.4386 (6)	0.04947 (14)	0.0345	
C7	0.44599 (12)	0.6265 (6)	0.08979 (13)	0.0319	
C8	0.42064 (11)	0.6495 (5)	0.14882 (12)	0.0236	
C9	0.43795 (11)	0.8470 (5)	0.18941 (13)	0.0273	
C10	0.43627 (11)	0.8646 (5)	0.25275 (13)	0.0278	
C11	0.41740 (11)	0.6923 (5)	0.29951 (12)	0.0248	
C12	0.43986 (12)	0.7061 (6)	0.36058 (13)	0.0312	
C13	0.42709 (13)	0.5401 (6)	0.40640 (14)	0.0363	
C14	0.39126 (14)	0.3563 (6)	0.39233 (13)	0.0362	
C15	0.36716 (12)	0.3447 (5)	0.33299 (13)	0.0291	
C16	0.37863 (11)	0.5125 (5)	0.28643 (12)	0.0225	
C17	0.19387 (11)	0.2237 (5)	0.72423 (12)	0.0211	
C18	0.15359 (11)	0.0105 (5)	0.72796 (11)	0.0217	
C19	0.11962 (11)	0.0120 (5)	0.78837 (12)	0.0227	
C20	0.13026 (12)	−0.1573 (5)	0.83494 (12)	0.0281	
C21	0.10561 (13)	−0.1443 (6)	0.89365 (13)	0.0342	
C22	0.07002 (13)	0.0420 (6)	0.90707 (14)	0.0361	
C23	0.05789 (12)	0.2069 (6)	0.86079 (13)	0.0305	
C24	0.08117 (11)	0.1940 (5)	0.80018 (12)	0.0241	
C25	0.06348 (11)	0.3688 (5)	0.75313 (13)	0.0270	
C26	0.06267 (11)	0.3504 (5)	0.68996 (13)	0.0277	
C27	0.08061 (11)	0.1544 (5)	0.64959 (12)	0.0235	
C28	0.05571 (11)	0.1337 (6)	0.58956 (13)	0.0301	
C29	0.06815 (12)	−0.0532 (6)	0.54957 (13)	0.0341	
C30	0.10567 (12)	−0.2249 (6)	0.56844 (13)	0.0300	
C31	0.13153 (12)	−0.2035 (5)	0.62664 (12)	0.0262	
C32	0.12027 (11)	−0.0156 (5)	0.66747 (12)	0.0221	
C33	0.24356 (17)	1.1206 (7)	0.52906 (16)	0.0547	
C34	0.20870 (16)	1.1411 (8)	0.47152 (17)	0.0612	
C35	0.26150 (18)	0.8437 (9)	0.42284 (16)	0.0654	
C36	0.29643 (16)	0.8250 (8)	0.47959 (16)	0.0576	
O2	0.20878 (8)	0.3034 (4)	0.67272 (9)	0.0319	
O1	0.29456 (9)	0.8317 (4)	0.27135 (9)	0.0416	
N1	0.28447 (10)	0.7810 (4)	0.16701 (10)	0.0276	
O4	0.26727 (11)	0.8892 (5)	0.53470 (11)	0.0601	
N2	0.21372 (10)	0.3049 (4)	0.77846 (10)	0.0301	
O3	0.23813 (13)	1.0729 (6)	0.41616 (11)	0.0766	
H12	0.2929	0.7005	0.1331	0.0337*	
H13	0.2388	0.4167	0.7778	0.0346*	
H14	0.2014	0.2492	0.8143	0.0341*	
H11	0.2604	0.8928	0.1658	0.0335*	
H21	0.3220	0.3680	0.2272	0.0227*	
H41	0.3422	0.1772	0.1371	0.0304*	
H51	0.3877	0.1358	0.0418	0.0373*	
H61	0.4516	0.4256	0.0100	0.0391*	
H71	0.4724	0.7421	0.0774	0.0366*	
H91	0.4521	0.9769	0.1669	0.0320*	
H101	0.4495	1.0070	0.2695	0.0308*	
H121	0.4646	0.8312	0.3704	0.0366*	
H131	0.4420	0.5553	0.4466	0.0428*	
H141	0.3833	0.2408	0.4229	0.0431*	
H151	0.3430	0.2207	0.3235	0.0324*	
H181	0.1771	−0.1295	0.7304	0.0252*	
H201	0.1550	−0.2818	0.8256	0.0321*	
H211	0.1138	−0.2613	0.9244	0.0401*	
H221	0.0540	0.0580	0.9478	0.0424*	
H231	0.0330	0.3320	0.8699	0.0339*	
H251	0.0491	0.5142	0.7700	0.0313*	
H261	0.0473	0.4794	0.6683	0.0327*	
H281	0.0308	0.2494	0.5766	0.0346*	
H291	0.0505	−0.0629	0.5088	0.0391*	
H301	0.1144	−0.3536	0.5422	0.0347*	
H311	0.1579	−0.3182	0.6393	0.0303*	
H331	0.2732	1.2396	0.5258	0.0635*	
H332	0.2202	1.1544	0.5657	0.0653*	
H341	0.1959	1.3056	0.4670	0.0711*	
H342	0.1775	1.0333	0.4757	0.0712*	
H351	0.2822	0.8002	0.3857	0.0752*	
H352	0.2310	0.7296	0.4268	0.0756*	
H361	0.3290	0.9332	0.4750	0.0676*	
H362	0.3100	0.6606	0.4851	0.0684*	

### Atomic displacement parameters (Å^2^)

**Table:**

	*U*^11^	*U*^22^	*U*^33^	*U*^12^	*U*^13^	*U*^23^
C1	0.0193 (15)	0.0282 (17)	0.0216 (15)	−0.0031 (12)	0.0011 (12)	−0.0002 (13)
C2	0.0250 (16)	0.0174 (14)	0.0214 (14)	−0.0029 (12)	0.0021 (11)	0.0015 (12)
C3	0.0228 (16)	0.0243 (16)	0.0199 (14)	0.0055 (12)	−0.0029 (11)	0.0048 (12)
C4	0.0286 (17)	0.0256 (16)	0.0232 (15)	0.0019 (13)	−0.0001 (12)	0.0003 (13)
C5	0.0371 (19)	0.0352 (19)	0.0250 (16)	0.0071 (15)	−0.0039 (13)	−0.0070 (14)
C6	0.0273 (17)	0.055 (2)	0.0216 (15)	0.0101 (16)	0.0024 (12)	−0.0003 (15)
C7	0.0238 (16)	0.043 (2)	0.0288 (16)	−0.0002 (14)	0.0043 (13)	0.0074 (15)
C8	0.0194 (15)	0.0283 (17)	0.0232 (15)	0.0031 (13)	−0.0003 (11)	0.0045 (13)
C9	0.0250 (16)	0.0251 (17)	0.0317 (17)	−0.0027 (13)	0.0000 (12)	0.0041 (14)
C10	0.0270 (16)	0.0234 (17)	0.0330 (17)	−0.0035 (13)	−0.0029 (13)	−0.0060 (14)
C11	0.0219 (15)	0.0273 (17)	0.0251 (15)	0.0055 (13)	0.0005 (12)	−0.0048 (13)
C12	0.0288 (17)	0.0322 (18)	0.0325 (17)	0.0017 (14)	−0.0046 (13)	−0.0073 (15)
C13	0.0364 (19)	0.048 (2)	0.0246 (16)	0.0080 (16)	−0.0066 (13)	−0.0019 (16)
C14	0.049 (2)	0.039 (2)	0.0212 (16)	0.0063 (16)	−0.0025 (14)	0.0051 (14)
C15	0.0314 (17)	0.0287 (18)	0.0274 (16)	−0.0003 (14)	0.0018 (12)	−0.0007 (14)
C16	0.0264 (16)	0.0234 (16)	0.0178 (14)	0.0052 (13)	0.0015 (11)	−0.0037 (12)
C17	0.0189 (15)	0.0242 (16)	0.0203 (15)	0.0013 (12)	−0.0009 (11)	−0.0041 (13)
C18	0.0228 (15)	0.0232 (16)	0.0190 (14)	0.0038 (12)	−0.0011 (11)	0.0007 (12)
C19	0.0216 (15)	0.0259 (16)	0.0207 (14)	−0.0044 (12)	−0.0013 (11)	−0.0032 (13)
C20	0.0337 (17)	0.0283 (17)	0.0224 (15)	0.0004 (14)	−0.0002 (12)	0.0015 (14)
C21	0.0423 (19)	0.040 (2)	0.0199 (15)	−0.0100 (16)	0.0002 (13)	0.0071 (14)
C22	0.0344 (18)	0.049 (2)	0.0244 (16)	−0.0085 (16)	0.0078 (13)	−0.0044 (16)
C23	0.0261 (17)	0.0343 (18)	0.0313 (17)	−0.0009 (14)	0.0046 (13)	−0.0086 (15)
C24	0.0242 (16)	0.0266 (16)	0.0215 (15)	−0.0038 (13)	0.0022 (12)	−0.0040 (13)
C25	0.0235 (16)	0.0228 (16)	0.0347 (17)	−0.0001 (12)	0.0048 (12)	−0.0026 (14)
C26	0.0268 (16)	0.0253 (17)	0.0310 (17)	0.0042 (13)	−0.0026 (13)	0.0048 (14)
C27	0.0221 (15)	0.0254 (17)	0.0231 (15)	−0.0036 (13)	0.0001 (11)	0.0018 (13)
C28	0.0227 (16)	0.0399 (19)	0.0277 (16)	−0.0010 (14)	−0.0035 (12)	0.0099 (15)
C29	0.0321 (18)	0.049 (2)	0.0207 (15)	−0.0079 (16)	−0.0020 (13)	−0.0008 (15)
C30	0.0341 (18)	0.0319 (18)	0.0241 (15)	−0.0064 (14)	0.0025 (13)	−0.0048 (13)
C31	0.0289 (17)	0.0266 (16)	0.0231 (15)	−0.0003 (13)	−0.0010 (12)	0.0017 (13)
C32	0.0195 (15)	0.0243 (16)	0.0225 (15)	−0.0037 (12)	0.0033 (11)	0.0039 (13)
C33	0.072 (3)	0.060 (3)	0.0321 (19)	−0.001 (2)	−0.0018 (18)	−0.0009 (19)
C34	0.059 (3)	0.075 (3)	0.049 (2)	−0.005 (2)	−0.0066 (19)	0.007 (2)
C35	0.069 (3)	0.097 (4)	0.031 (2)	0.008 (3)	−0.0032 (18)	−0.009 (2)
C36	0.057 (3)	0.070 (3)	0.046 (2)	−0.009 (2)	0.0046 (18)	0.002 (2)
O2	0.0365 (13)	0.0357 (12)	0.0235 (11)	−0.0099 (10)	0.0011 (9)	0.0034 (10)
O1	0.0443 (14)	0.0513 (15)	0.0292 (12)	0.0234 (12)	−0.0061 (10)	−0.0082 (11)
N1	0.0272 (14)	0.0302 (14)	0.0256 (13)	0.0092 (11)	−0.0009 (10)	−0.0014 (11)
O4	0.0769 (19)	0.073 (2)	0.0303 (13)	0.0053 (16)	−0.0001 (12)	0.0113 (13)
N2	0.0307 (14)	0.0366 (15)	0.0231 (13)	−0.0155 (12)	0.0048 (10)	−0.0024 (11)
O3	0.088 (2)	0.109 (3)	0.0327 (15)	0.010 (2)	−0.0051 (14)	0.0206 (16)

### Geometric parameters (Å, °)

**Table:**

C1—C2	1.539 (4)	C20—H201	0.940
C1—O1	1.232 (3)	C21—C22	1.382 (4)
C1—N1	1.320 (3)	C21—H211	0.944
C2—C3	1.508 (4)	C22—C23	1.375 (4)
C2—C16	1.511 (4)	C22—H221	0.948
C2—H21	0.981	C23—C24	1.401 (4)
C3—C4	1.385 (4)	C23—H231	0.944
C3—C8	1.408 (4)	C24—C25	1.457 (4)
C4—C5	1.381 (4)	C25—C26	1.337 (4)
C4—H41	0.946	C25—H251	0.955
C5—C6	1.382 (4)	C26—C27	1.457 (4)
C5—H51	0.945	C26—H261	0.932
C6—C7	1.384 (4)	C27—C28	1.404 (4)
C6—H61	0.940	C27—C32	1.400 (4)
C7—C8	1.397 (4)	C28—C29	1.379 (4)
C7—H71	0.946	C28—H281	0.924
C8—C9	1.460 (4)	C29—C30	1.378 (4)
C9—C10	1.341 (4)	C29—H291	0.959
C9—H91	0.935	C30—C31	1.379 (4)
C10—C11	1.456 (4)	C30—H301	0.934
C10—H101	0.929	C31—C32	1.389 (4)
C11—C12	1.397 (4)	C31—H311	0.942
C11—C16	1.400 (4)	C33—C34	1.477 (5)
C12—C13	1.378 (4)	C33—O4	1.423 (4)
C12—H121	0.943	C33—H331	0.980
C13—C14	1.376 (4)	C33—H332	0.977
C13—H131	0.924	C34—O3	1.422 (4)
C14—C15	1.379 (4)	C34—H341	0.976
C14—H141	0.934	C34—H342	0.969
C15—C16	1.389 (4)	C35—C36	1.464 (5)
C15—H151	0.928	C35—O3	1.410 (5)
C17—C18	1.542 (4)	C35—H351	0.963
C17—O2	1.231 (3)	C35—H352	0.979
C17—N2	1.319 (3)	C36—O4	1.409 (4)
C18—C19	1.519 (4)	C36—H361	0.997
C18—C32	1.511 (3)	C36—H362	0.984
C18—H181	0.969	N1—H12	0.871
C19—C20	1.389 (4)	N1—H11	0.855
C19—C24	1.401 (4)	N2—H13	0.870
C20—C21	1.379 (4)	N2—H14	0.873
			
C2—C1—O1	120.0 (2)	C20—C21—H211	119.5
C2—C1—N1	117.9 (2)	C22—C21—H211	120.8
O1—C1—N1	122.0 (3)	C21—C22—C23	119.5 (3)
C1—C2—C3	113.0 (2)	C21—C22—H221	120.9
C1—C2—C16	109.8 (2)	C23—C22—H221	119.6
C3—C2—C16	115.6 (2)	C22—C23—C24	122.0 (3)
C1—C2—H21	105.5	C22—C23—H231	119.2
C3—C2—H21	106.1	C24—C23—H231	118.9
C16—C2—H21	106.0	C19—C24—C23	117.9 (3)
C2—C3—C4	119.3 (3)	C19—C24—C25	124.0 (2)
C2—C3—C8	121.3 (2)	C23—C24—C25	118.0 (3)
C4—C3—C8	119.3 (2)	C24—C25—C26	129.0 (3)
C3—C4—C5	121.5 (3)	C24—C25—H251	115.1
C3—C4—H41	119.0	C26—C25—H251	115.8
C5—C4—H41	119.5	C25—C26—C27	129.7 (3)
C4—C5—C6	119.8 (3)	C25—C26—H261	115.7
C4—C5—H51	119.2	C27—C26—H261	114.6
C6—C5—H51	121.0	C26—C27—C28	117.6 (3)
C5—C6—C7	119.3 (3)	C26—C27—C32	124.0 (2)
C5—C6—H61	120.0	C28—C27—C32	118.3 (3)
C7—C6—H61	120.7	C27—C28—C29	121.5 (3)
C6—C7—C8	121.9 (3)	C27—C28—H281	118.9
C6—C7—H71	119.4	C29—C28—H281	119.7
C8—C7—H71	118.8	C28—C29—C30	119.9 (3)
C3—C8—C7	118.1 (3)	C28—C29—H291	119.7
C3—C8—C9	124.0 (2)	C30—C29—H291	120.4
C7—C8—C9	118.0 (3)	C29—C30—C31	119.3 (3)
C8—C9—C10	129.1 (3)	C29—C30—H301	121.2
C8—C9—H91	113.3	C31—C30—H301	119.6
C10—C9—H91	117.6	C30—C31—C32	122.0 (3)
C9—C10—C11	129.7 (3)	C30—C31—H311	119.5
C9—C10—H101	115.4	C32—C31—H311	118.5
C11—C10—H101	114.9	C18—C32—C27	121.4 (2)
C10—C11—C12	117.9 (3)	C18—C32—C31	119.5 (2)
C10—C11—C16	123.7 (2)	C27—C32—C31	119.0 (2)
C12—C11—C16	118.4 (3)	C34—C33—O4	111.5 (3)
C11—C12—C13	121.5 (3)	C34—C33—H331	107.5
C11—C12—H121	119.0	O4—C33—H331	109.5
C13—C12—H121	119.4	C34—C33—H332	107.9
C12—C13—C14	119.8 (3)	O4—C33—H332	110.1
C12—C13—H131	119.7	H331—C33—H332	110.3
C14—C13—H131	120.5	C33—C34—O3	111.8 (3)
C13—C14—C15	119.5 (3)	C33—C34—H341	109.3
C13—C14—H141	120.1	O3—C34—H341	109.4
C15—C14—H141	120.4	C33—C34—H342	108.3
C14—C15—C16	121.7 (3)	O3—C34—H342	107.3
C14—C15—H151	119.5	H341—C34—H342	110.8
C16—C15—H151	118.8	C36—C35—O3	112.0 (3)
C2—C16—C11	121.4 (2)	C36—C35—H351	110.5
C2—C16—C15	119.6 (3)	O3—C35—H351	111.0
C11—C16—C15	118.9 (2)	C36—C35—H352	108.2
C18—C17—O2	120.9 (2)	O3—C35—H352	107.7
C18—C17—N2	116.7 (2)	H351—C35—H352	107.2
O2—C17—N2	122.3 (3)	C35—C36—O4	111.7 (3)
C17—C18—C19	112.4 (2)	C35—C36—H361	109.1
C17—C18—C32	111.3 (2)	O4—C36—H361	108.7
C19—C18—C32	115.1 (2)	C35—C36—H362	110.6
C17—C18—H181	105.2	O4—C36—H362	108.0
C19—C18—H181	106.1	H361—C36—H362	108.7
C32—C18—H181	105.9	C1—N1—H12	120.5
C18—C19—C20	119.5 (3)	C1—N1—H11	118.2
C18—C19—C24	120.9 (2)	H12—N1—H11	121.2
C20—C19—C24	119.4 (2)	C33—O4—C36	111.5 (3)
C19—C20—C21	121.4 (3)	C17—N2—H13	118.9
C19—C20—H201	118.2	C17—N2—H14	120.4
C21—C20—H201	120.4	H13—N2—H14	120.7
C20—C21—C22	119.7 (3)	C34—O3—C35	111.3 (3)

### Hydrogen-bond geometry (Å, °)

**Table:**

*D*—H···*A*	*D*—H	H···*A*	*D*···*A*	*D*—H···*A*
N1—H11···O2^i^	0.85	2.11	2.962 (3)	171
N1—H12···O4^i^	0.87	2.22	2.978 (3)	145
N2—H13···O1^ii^	0.87	1.95	2.823 (3)	177
N2—H14···O3^ii^	0.87	2.53	3.040 (3)	119

Symmetry codes: (i) *x*, −*y*+3/2, *z*−1/2; (ii) *x*, −*y*+3/2, *z*+1/2.
